# New daily persistent headache after SARS-CoV-2 infection in Latin America: a cross-sectional study

**DOI:** 10.1186/s12879-023-08898-2

**Published:** 2023-12-14

**Authors:** Fhabián S. Carrión-Nessi, Luis C. Ascanio, Andreína G. Pineda-Arapé, Óscar D. Omaña-Ávila, Daniela L. Mendoza-Millán, Sinibaldo R. Romero, Abranny B. Almao-Rivero, Natasha A. Camejo-Ávila, Karim J. Gebran-Chedid, Carlis M. Rodriguez-Saavedra, Diana C. Freitas-De Nobrega, Sergio A. Castañeda, José L. Forero-Peña, Lourdes A. Delgado-Noguera, Lucianny K. Meneses-Ramírez, Juan C. Cotuá, Alfonso J. Rodriguez-Morales, David A. Forero-Peña, Alberto E. Paniz-Mondolfi

**Affiliations:** 1 Biomedical Research and Therapeutic Vaccines Institute, Ciudad Bolivar, Venezuela; 2 Department of Infectious Diseases and Tropical Medicine, Venezuelan Science Incubator, Barquisimeto, Venezuela; 3 “Luis Razetti” School of Medicine, Central University of Venezuela, Caracas, Venezuela; 4 Department of Pathology, Molecular and Cell-Based Medicine, Icahn School of Medicine at Mount Sinai, New York City, NY USA; 5 Medical Scientist Training Program (MD/PhD), University of Minnesota Medical School, Minneapolis, MN USA; 6 Centro de Investigaciones en Microbiología y Biotecnología, Facultad de Ciencias Naturales, Universidad del Rosario (CIMBIUR), Universidad del Rosario, Bogotá, Colombia; 7 “Dr. Francisco Battistini Casalta” Health Sciences School, University of Oriente – Bolivar Nucleus, Ciudad Bolivar, Venezuela; 8 Gilbert and Rose-Marie Chagoury School of Medicine, Lebanese American University, Beirut, Lebanon; 9 Master of Clinical Epidemiology and Biostatistics, Universidad Científica del Sur, Lima, Peru; 10 Infectious Diseases Department, University Hospital of Caracas, Caracas, Venezuela

**Keywords:** New daily persistent headache, NDPH, SARS-CoV-2, COVID-19, Long COVID, Latin America, Cross-sectional survey

## Abstract

**Background:**

Persistent headache is a frequent symptom after coronavirus disease 2019 (COVID-19) and there is currently limited knowledge about its clinical spectrum and predisposing factors. A subset of patients may be experiencing new daily persistent headache (NDPH) after COVID-19, which is among the most treatment-refractory primary headache syndromes.

**Methods:**

We conducted a cross-sectional study in Latin America to characterize individuals with persistent headache after severe acute respiratory syndrome coronavirus 2 (SARS-CoV-2) infection and to identify factors associated with NDPH. Participants over 18 years old who tested positive for SARS-CoV-2 infection and reported persistent headache among their symptoms completed an online survey that included demographics, past medical history, persistent headache clinical characteristics, and COVID-19 vaccination status. Based on participants’ responses, NDPH diagnostic criteria were used to group participants into NDPH and non-NDPH groups. Participant data was summarized by descriptive statistics. Student’s t and Mann–Whitney U tests were used according to the distribution of quantitative variables. For categorical variables, Pearson’s chi-square and Fisher’s exact tests were used according to the size of expected frequencies. Binomial logistic regression using the backward stepwise selection method was performed to identify factors associated with NDPH.

**Results:**

Four hundred and twenty-one participants from 11 Latin American countries met the inclusion criteria. One in four participants met the NDPH diagnostic criteria. The mean age was 40 years, with most participants being female (82%). Over 90% of the participants reported having had mild/moderate COVID-19. Most participants had a history of headache before developing COVID-19 (58%), mainly migraine type (32%). The most predominant clinical characteristics in the NDPH group were occipital location, severe/unbearable intensity, burning character, and radiating pain (*p* < 0.05). A higher proportion of anxiety symptoms, sleep problems, myalgia, mental fog, paresthesia, nausea, sweating of the face or forehead, and ageusia or hypogeusia as concomitant symptoms were reported in participants with NDPH (*p* < 0.05). Palpebral edema as a concomitant symptom during the acute phase of COVID-19, occipital location, and burning character of the headache were risk factors associated with NDPH.

**Conclusion:**

This is the first study in Latin America that explored the clinical spectrum of NDPH after SARS-CoV-2 infection and its associated factors. Clinical evaluation of COVID-19 patients presenting with persistent headache should take into consideration NDPH.

**Supplementary Information:**

The online version contains supplementary material available at 10.1186/s12879-023-08898-2.

## Background

As of December 13, 2023, coronavirus disease 2019 (COVID-19) has affected over 772 million people and caused more than 6.9 million deaths worldwide [1]. A significant number of patients recovering from acute COVID-19 infection experience persistent symptoms for weeks or months, including fatigue, dyspnea, myalgia, muscle weakness, and depression. Neurological symptoms, such as mental fog with cognitive disturbances and persistent headache, are expected during the acute phase of the disease [2–6] and may persist for more than six months [7, 8].

Persistent headache, with a prevalence ranging from 8 to 15% in the first six months after COVID-19 remission, is a frequent symptom [9]. However, limited knowledge exists regarding the clinical spectrum and predisposing factors of persistent headache following COVID-19 [10–12]. Some patients with persistent headache had a pre-existing primary headache syndrome, and that severe acute respiratory syndrome coronavirus 2 (SARS-CoV-2) infection may have triggered exacerbation or chronicity of the cephalic syndrome [13, 14]. However, a subgroup may develop new daily persistent headache (NDPH), a treatment-refractory primary headache syndrome [12, 15].

NDPH is characterized as a daily headache, well-defined from onset, clearly remembered, with pain becoming continuous and unremitting within 24 h and persisting for more than three months in individuals without a previous history of headache [16]. However, some patients develop NDPH within two weeks of COVID-19 resolution, regardless of past headache history. NDPH may have features suggestive of migraine, tension headache, or both [16]. It is usually bilateral, from moderate to severe intensity, and sometimes associated with nausea, vomiting, or photophobia. Although there are no known factors related to NDPH development, viral infections, including SARS-CoV-2, may trigger it [17]. Despite this, there is still limited information about this topic [15, 18, 19], particularly in Latin America. This study aims to characterize individuals with persistent headache after SARS-CoV-2 infection in Latin America, particularly NDPH, and identify potential factors associated with NDPH.

## Methods

### Study design

**Fig. 1:**
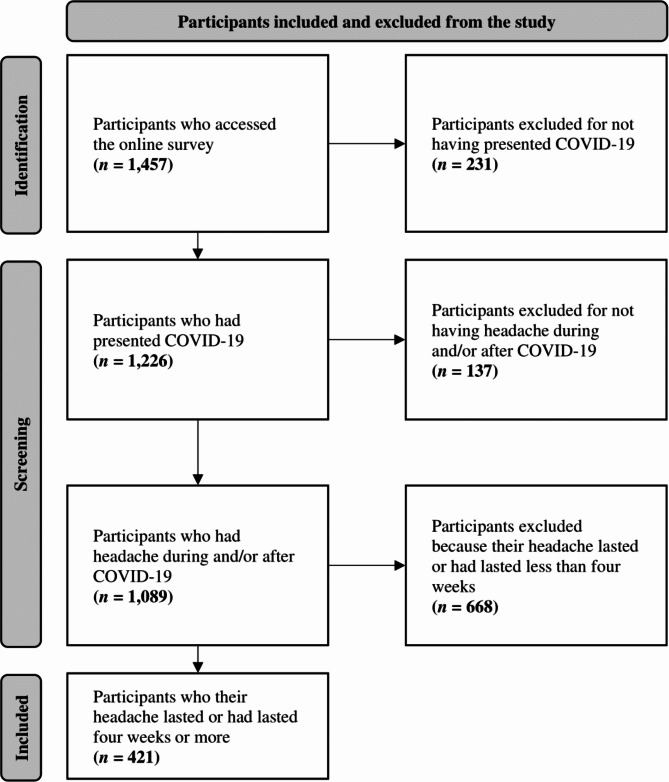
Flowchart of the participants’ selection

An online cross-sectional survey was conducted in 11 Latin American countries between April 15 and 30, 2022 using the “Google Forms” platform (Google LLC, Mountain View, CA, USA). Participants over 18 years old who tested positive for SARS-CoV-2 infection based on reverse transcriptase polymerase chain reaction (RT-PCR) or antigen testing [20] and who had persistent headache (for more than 28 days) [21] among their symptoms were included. Surveys reporting inconsistent/incomplete responses on conditional items (3, 4, 9, 5, 8, 9, 13, 14, 18, 19, 31, 38, 39, 40, 50, 51 and 52 in Supplementary Data 1) were excluded. The survey was designed to screen potential participants and automatically exclude those not meeting inclusion criteria, ending the survey before completion (Fig. 1).

Participants were invited using the instant messaging application WhatsApp and emails via a survey link. Also, fliers with the survey link were posted on prominent social media accounts of Latin American national and international health-related institutions/guilds/societies. The survey was voluntary, anonymous, and confidential.

### Sample size

According to the Pan American Health Organization [22], by April 12, 2022, there were 62,653,758 confirmed SARS-CoV-2 cases in Latin America. Hence, the sample size, with a 95% confidence interval and a 5% margin of error, was at least 384 participants. The sampling method was non-probabilistic.

### Survey design and data collection

A detailed survey focused on the characteristics of persistent headache, in addition to some neurological symptoms of COVID-19, after rounds of online discussions among the authors was designed. The survey was in Spanish and consisted of 37 questions distributed among four different sections assessing demographics, past medical history, persistent headache clinical characteristics, and COVID-19 vaccination status (Supplementary Data 1). Demographics included age, sex, education level, marital status, race, occupation, and country of residence. Past medical history data included COVID-19 clinical presentation, comorbidities, smoking habits, personal and family history of headache before the COVID-19 pandemic, and neurological symptoms during acute COVID-19. Persistent headache clinical characteristics included onset, side, location, intensity, character, irradiation, duration, onset schedule, pain attenuation (reduction or weakening of the perception of pain due to analgesics, rest, or sleep), pain exacerbation (increase or intensification in the severity of pain due to Valsalva maneuvers, head movement, or walking), and other concomitant symptoms. Pain intensity was assessed with the Visual Analogue Scale (VAS). COVID-19 vaccination status data included vaccination status before headache onset, type of vaccine, vaccination scheme, and time from last dose to headache onset.

### Survey validation and pilot test

Infectious disease specialists, neurologists, and epidemiologists assessed and validated the survey. A pilot survey was tested on 30 participants, with a mean age of 33 (SD —standard deviation— 11) years and predominantly female (*n* = 20, 66.7%), to check for questions’ clarity one month before its application.

### NDPH diagnostic criteria

NDPH was first documented in 1986 [23]. Previously known as chronic headache with acute onset or *de novo* chronic headache, it is a primary headache characterized by persistent pain, daily from its onset, which is clearly remembered. The pain lacks characteristic features and may exhibit migraine-like or tension-type-like attributes or combine elements of both. According to the third edition of the International Classification of Headache Disorders (ICHD-3), the NDPH diagnostic criteria are as follows [16]:

A. Persistent headache fulfilling criteria B and C.
B. Distinct and clearly-remembered onset, with pain becoming continuous and unremitting within 24 h.
C. Present for > 3 months.
D. Not better accounted for by another ICHD-3 diagnosis.

However, if persistent headache fulfills criteria B but is present for ≤ 3 months and is not better accounted for by another ICHD-3 diagnosis, it is classified as a probable NDPH [16]. Based on the participants’ responses to survey items 3, 17, 18, 19, 26 and 27 (Supplementary Data 1), those fulfilling the diagnostic criteria for NDPH and probable NDPH were separated from the rest. All participants who met these criteria (NDPH or probable NDPH) were grouped as NDPH. The rest were grouped as non-NDPH.

### Statistical analysis

Participant data was summarized by mean, standard deviation (SD), median, interquartile range (IQR), and/or frequency, percentage (%). The Kolmogorov–Smirnov test was used to assess the normal distribution of numeric variables. Univariable analyses were performed as follows: Mann–Whitney U test for numerical variables with a non-normal distribution and Student’s t-test for those with normal distribution. Pearson’s chi-squared and Fisher’s exact tests were used for categorical variables according to the size of expected frequencies. When needed, a *post-hoc* analysis was performed using the Bonferroni correction of the *p* value to adjust it. *P* values < 0.05 were considered significant. Statistically significant variables identified in the univariable analyses were included in a binomial logistic regression using a backward stepwise selection method to identify the factors associated with NDPH. For this analysis, associated factors that belonged to a broader group were grouped and coded as “yes” or “no”. A “yes” indicated the presence of the associated factor, while a “no” indicated the presence of any other associated factor. The “no” response was used as a reference for comparison. The best valid model that classified the highest percentage of participants, including the goodness of fit, the R^2^ Nagelkerke, and the Hosmer–Lemeshow test was considered. Statistical analyses were performed using SPSS version 26 (IBM Corporation, Armonk, NY, USA). Figures were generated using Microsoft® Excel® and PowerPoint® version 2019 (Microsoft, Redmond, WA, USA).

## Results

### Participants’ demographics

**Fig. 2:**
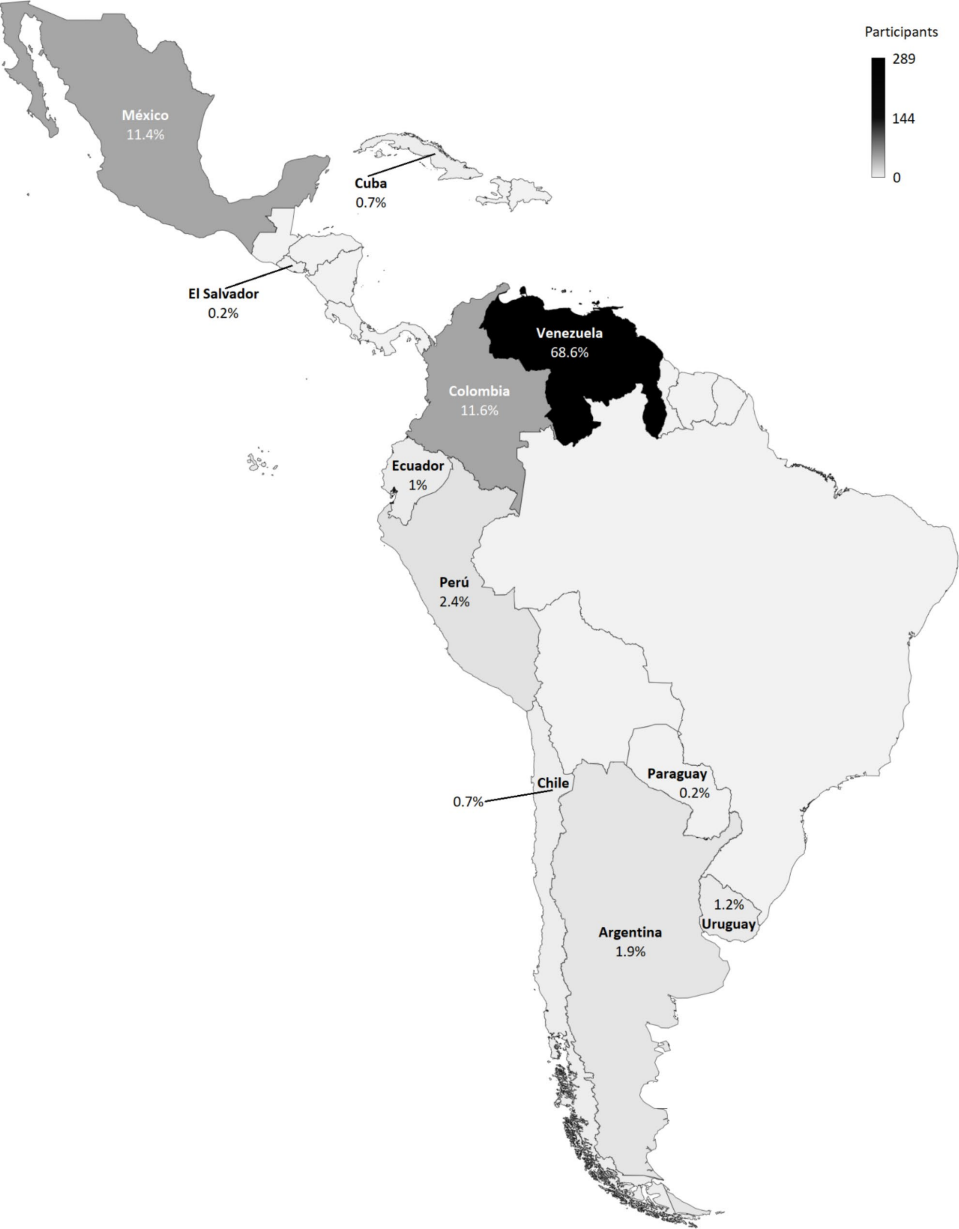
Participants from 11 Latin American countries that met the inclusion criteria. The number of participants surveyed is represented in grayscale. The frequency of participants surveyed is represented as a percentage within each country

A total of 421 participants (Fig. 1) from 11 Latin American countries (Fig. 2) met the inclusion criteria. Out of these, 106 (25.2%) met the diagnostic criteria for NDPH: 60 (56.6%) for NDPH and 46 (43.4%) for probable NDPH. The mean age was 40 (SD 12, range: 18–82) years; most were female (81.5%, *n* = 343), with higher level (university) education (76.2%, *n* = 321), and self-perceived as of mixed race (50.4%, *n* = 212). There were no statistically significant differences between the demographics of participants with NDPH and non-NDPH (Table 1). Among participants who worked or studied (*n* = 391), the majority reported working daytime hours (62.9%, *n* = 246/391) and for more than eight hours (56%, *n* = 219/391).

**Table 1:** Demographics of 421 participants with persistent headache after COVID-19 in Latin America

Demographics	All (*n* = 421, 100%)	NDPH (*n* = 106, 25.2%)	Non-NDPH (*n* = 315, 74.8%)	*p* value
Age, mean (SD), years	40 (12)	41 (13)	39 (12)	0.139^a^
Sex, *n* (%)				0.636^b^
Female	343 (81.5)	88 (83)	255 (81)	
Male	78 (18.5)	18 (17)	60 (19)	
Education level, *n* (%)				0.132^c^
None	1 (0.2)	0 (0)	1 (0.3)	
Primary school	3 (0.7)	2 (1.9)	1 (0.3)	
High school	44 (10.5)	7 (6.6)	37 (11.7)	
Associate degree	52 (12.4)	10 (9.4)	42 (13.3)	
University	321 (76.2)	87 (82.1)	234 (74.3)	
Marital status, *n* (%)				0.077^b^
Single	167 (39.7)	35 (33)	132 (41.9)	
Married	164 (39)	39 (36.8)	125 (39.7)	
Common-law marriage	59 (14)	19 (17.9)	40 (12.7)	
Divorced	21 (5)	8 (7.5)	13 (4.1)	
Widowed	10 (2.4)	5 (4.7)	5 (1.6)	
Race, *n* (%)				0.374^c^
Mixed	212 (50.4)	51 (48.1)	161 (51.1)	
White	196 (46.6)	51 (48.1)	145 (46)	
Black	9 (2.1)	4 (3.8)	5 (1.6)	
Indigenous	4 (1)	0 (0)	4 (1.3)	
Occupation, *n* (%)				0.241^b^
Employee	140 (33.3)	37 (34.9)	103 (32.7)	
Healthcare worker	137 (32.5)	29 (27.4)	108 (34.3)	
Self-employed	74 (17.6)	25 (23.6)	49 (15.6)	
Student	40 (9.5)	7 (6.6)	33 (10.5)	
Unemployed/Retired	30 (7.1)	8 (7.5)	22 (7)	

^a^Student’s t-test for independent samples; ^b^Pearson’s chi-square test; ^c^Fisher’s exact test

### Past medical history

While over 90% of the participants reported having had mild/moderate COVID-19, there were no differences in COVID-19 clinical presentation (*p* = 0.889). Hypertension (21.6%, *p* = 0.767) was the most common comorbidity, followed by asthma (8.3%, *p* = 0.939), diabetes (3.3%, *p* = 0.356), and hypothyroidism (3.1%, *p* = 0.409). None of the comorbidities nor smoking habits were different between the two groups. However, participants within the NDPH group had a higher pack-year index (4.6 vs. 0.8, *p* = 0.039). Most participants had a personal history of headache before developing COVID-19 (58.4%, *n* = 246), mainly migraine type (32.3%), with no statistically significant differences between the two groups (*p* = 0.673) (Table 2).

**Table 2:** Past medical history of 421 participants with persistent headache after COVID-19 in Latin America

Past medical history	All (*n* = 421, 100%)	NDPH (*n* = 106, 25.2%)	Non-NDPH (*n* = 315, 74.8%)	*p* value
COVID-19 clinical presentation, *n* (%)				0.889^a^
Mild/Moderate	396 (94.1)	100 (94.3)	296 (94)	
Severe/Critical	25 (5.9)	6 (5.7)	19 (6)	
Comorbidities, yes (%)				
Hypertension	91 (21.6)	24 (22.6)	67 (21.3)	0.767^a^
Asthma	35 (8.3)	9 (8.5)	26 (8.3)	0.939^a^
Diabetes	14 (3.3)	5 (4.7)	9 (2.9)	0.356^a^
Hypothyroidism	13 (3.1)	2 (1.9)	11 (3.5)	0.409^a^
HIV	4 (1)	0 (0)	4 (1.3)	0.576^b^
Cancer	4 (1)	3 (2.8)	1 (0.3)	0.051^b^
COPD	2 (0.5)	2 (1.9)	0 (0)	0.063^b^
CKD	1 (0.2)	1 (0.9)	0 (0)	0.252^b^
Smoking habits, *n* (%)				0.232^a^
No	392 (93.1)	96 (90.6)	296 (94)	
Yes	29 (6.9)	10 (9.4)	19 (6)	
Pack-year index, median (IQR)	1.5 (5.5)	4.6 (9)	0.8 (5)	0.039^c^
Personal history of headache before COVID-19 pandemic, *n* (%)				0.261^a^
No	175 (41.6)	49 (46.2)	126 (40)	
Yes	246 (58.4)	57 (53.8)	189 (60)	
Migraine, yes (%)	136 (32.3)	36 (34)	100 (31.7)	0.673^a^
Tension, yes (%)	100 (23.8)	20 (18.9)	80 (25.4)	0.172^a^
Other, yes (%)	29 (6.9)	9 (8.5)	20 (6.3)	0.451^a^
Family history of headache before COVID-19 pandemic, *n* (%)				0.722^a^
No	256 (60.8)	66 (62.3)	190 (60.3)	
Yes	165 (39.2)	40 (37.7)	125 (39.7)	
Migraine, yes (%)	130 (30.9)	31 (29.2)	99 (31.4)	0.674^a^
Tension, yes (%)	43 (10.2)	11 (10.4)	32 (10.2)	0.949^a^

^a^Pearson’s chi-square test; ^b^Fisher’s exact test; ^c^Mann-Whitney U test. HIV: human immunodeficiency virus; COPD: chronic obstructive pulmonary disease; CKD: chronic kidney disease

### Concomitant symptoms during the acute phase of COVID-19

Fatigue (81.7%, *n* = 344), myalgia (62.5%, *n* = 263), sleep disorders (53.7%, *n* = 226), anxiety symptoms (49.2%, *n* = 207), and mental fog (47%, *n* = 198) were the most frequent concomitant symptoms during the acute phase of COVID-19. There was a higher proportion of sweating of the face or forehead, drooping of the upper eyelid and/or pupillary constriction, and palpebral edema in participants with NDPH (34.9% vs. 22.2%, *p* = 0.009; 6.6% vs. 1.3%, *p* = 0.003; 14.2% vs. 2.5%, *p* < 0.001; respectively).

### Persistent headache clinical characteristics

Clinical characteristics are summarized in Table 3. Persistent headache began during the first two weeks of COVID-19 in most participants (68.9%). Among participants with persistent headache < 3 months (50.8%, *n* = 214), the majority (40.2%, *n* = 86/214) reported having had it for 4–8 weeks, while among participants with persistent headache ≥ 3 months (49.2%, *n* = 207), the majority (39.1%, *n* = 81/207) reported having it for > 12 months. The most predominant clinical characteristics were occipital location (43.4% vs. 28.3%, *p* = 0.004), severe/unbearable intensity (70.8% vs. 56.8%, *p* = 0.011), burning character (17% vs. 6.7%, *p* = 0.002), and radiating pain (70.8% vs. 60%, *p* = 0.048). Conversely, there was a higher proportion of attenuating pain found in participants with non-NDPH compared to NDPH (96.5% vs. 89.6%, *p* = 0.006).

**Table 3:** Persistent headache clinical characteristics after COVID-19 in 421 participants in Latin America

Persistent headache clinical characteristics	All (*n* = 421, 100%)	NDPH (*n* = 106, 25.2%)	Non-NDPH (*n* = 315, 74.8%)	*p* value
Time of onset, *n* (%)				0.744^a^
< 2nd week of COVID-19	290 (68.9)	70 (66)	220 (69.8)	
2nd − 4th week of COVID-19	64 (15.2)	17 (16)	47 (14.9)	
> 4th week of COVID-19	67 (15.9)	19 (17.9)	48 (15.2)	
Type of onset, *n* (%)				0.476^a^
Insidious	278 (66)	73 (68.9)	205 (65.1)	
Sudden	143 (34)	33 (31.1)	110 (34.9)	
Side, *n* (%)				0.472^a^
Bilateral	286 (67.9)	75 (70.8)	211 (67)	
Unilateral	135 (32.1)	31 (29.2)	104 (33)	
Location, yes (%)				
Frontal	212 (50.4)	51 (48.1)	161 (51.1)	0.593^a^
Parietal	165 (39.2)	41 (38.7)	124 (39.4)	0.9^a^
Periocular	163 (38.7)	48 (45.3)	115 (36.5)	0.109^a^
Occipital	135 (32.1)	46 (43.4)	89 (28.3)	0.004^a^
Temporal	106 (25.2)	24 (22.6)	82 (26)	0.487^a^
Vertex	74 (17.6)	20 (18.9)	54 (17.1)	0.686^a^
Intensity (VAS) score, median (IQR), points	7 (3)	8 (2)	7 (3)	0.007^b^
Intensity (VAS) by severity, *n* (%)				0.011^a^
Mild/Moderate	167 (39.7)	31 (29.2)	136 (43.2)	
Severe/Unbearable	254 (60.3)	75 (70.8)	179 (56.8)	
Character, yes (%)				
Oppressive	248 (58.9)	63 (59.4)	185 (58.7)	0.899^a^
Pulsatile	167 (39.7)	48 (45.3)	119 (37.8)	0.172^a^
Dull	120 (28.5)	28 (26.4)	92 (29.2)	0.582^a^
Lancinating	111 (26.4)	34 (32.1)	77 (24.4)	0.123^a^
Burning	39 (9.3)	18 (17)	21 (6.7)	0.002^a^
Irradiation, *n* (%)				0.048^a^
No	157 (37.3)	31 (29.2)	126 (40)	
Yes	264 (62.7)	75 (70.8)	189 (60)	
Neck, yes (%)	162 (38.5)	49 (46.2)	113 (35.9)	0.058^a^
Face, yes (%)	126 (29.9)	38 (35.8)	88 (27.9)	0.124^a^
Shoulders, yes (%)	97 (23)	30 (28.3)	67 (21.3)	0.137^a^
Other, yes (%)	4 (1)	3 (2.8)	1 (0.3)	0.051^c^
Duration, *n* (%)				0.077^a^
< 3 months	214 (50.8)	46 (43.4)	168 (53.3)	0.01^a^
4–8 weeks	86 (40.2)	15 (32.6)	71 (42.3)	
5–6 weeks	26 (12.1)	4 (8.7)	22 (13.1)	
6–7 weeks	16 (7.5)	0 (0)	16 (9.5)	
7–8 weeks	28 (13.1)	6 (13)	22 (13.1)	
8 weeks to 3 months	58 (13.1)	21 (45.7)	37 (22)	
≥ 3 months	207 (49.2)	60 (56.6)	147 (46.7)	0.035^a^
3–6 months	64 (30.9)	25 (41.7)	39 (26.5)	
6–9 months	38 (18.4)	14 (23.3)	24 (16.3)	
9–12 months	24 (11.6)	4 (6.7)	20 (13.6)	
> 12 months	81 (39.1)	17 (28.3)	64 (43.5)	
Onset schedule, *n* (%)				0.004^a^
Any time of day	243 (57.7)	71 (67)	172 (54.6)	
During the morning	81 (19.2)	18 (17)	63 (20)	
During the afternoon	57 (13.6)	4 (3.8)	53 (16.8)	
During the evening	40 (9.5)	13 (12.3)	27 (8.6)	
Pain attenuation, *n* (%)				0.006^a^
No	22 (5.2)	11 (10.4)	11 (3.5)	
Yes	399 (94.8)	95 (89.6)	304 (96.5)	
Analgesics, yes (%)	340 (80.8)	79 (74.5)	261 (82.9)	0.06^a^
Rest, yes (%)	114 (27.1)	25 (23.6)	89 (28.3)	0.349^a^
Sleep, yes (%)	109 (25.9)	24 (22.6)	85 (27)	0.377^a^
Pain exacerbation, *n* (%)				0.553^a^
No	83 (19.7)	23 (21.7)	60 (19)	
Yes	338 (80.3)	83 (78.3)	255 (81)	
Valsalva maneuvers, yes (%)	190 (45.1)	49 (46.2)	141 (44.8)	0.793^a^
Head movements, yes (%)	177 (42)	38 (35.8)	139 (44.1)	0.135^a^
Walking, yes (%)	78 (18.5)	25 (23.6)	53 (16.8)	0.121^a^

^a^Pearson’s chi-square test; ^b^Mann-Whitney U test; ^c^Fisher’s exact test

**Fig. 3:**
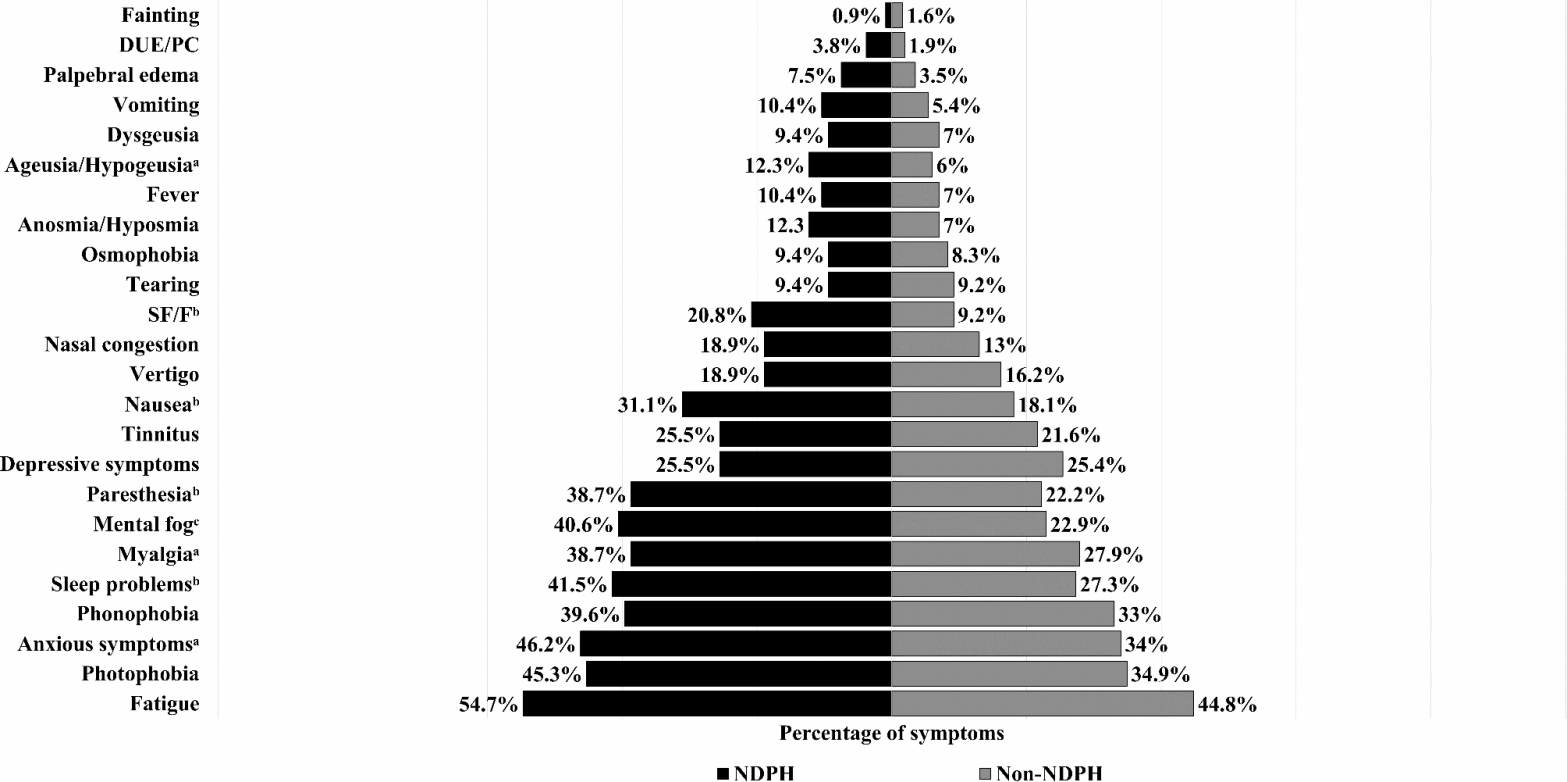
Concomitant symptoms of persistent headache after COVID-19 of 421 participants in Latin America. Data are plotted as percentage. ^a^*p* < 0.05; ^b^*p* < 0.01; ^c^*p* < 0.001 (*p* values by Pearson’s chi-square test). SF/F: sweating of the face or forehead; DUE/PC: drooping of the upper eyelid and/or pupillary constriction

Overall, Fatigue (47.3%, *n* = 199), photophobia (37.5%, *n* = 158), anxiety symptoms (37.1%, *n* = 156), phonophobia (34.7%, *n* = 146), and sleep disorders (30.9%, *n* = 130) were the most frequent concomitant symptoms of persistent headache. There was a significantly higher proportion of anxious symptoms (46.2% vs. 34%), sleep problems (41.5% vs. 27.3%), myalgia (38.7% vs. 27.9%), mental fog (40.6% vs. 22.9%), paresthesia (38.7% vs. 22.2%), nausea (31.1% vs. 18.1%), sweating of the face or forehead (20.8% vs. 9.2%), and ageusia or hypogeusia (12.3% vs. 6%) as concomitant symptoms in participants with NDPH compared with non-NDPH (Fig. 3).

### COVID-19 vaccination status

Most participants were vaccinated against COVID-19 before developing persistent headache (60.3%, *n* = 254), with no differences between the two groups (*p* = 0.827). BBIBP-CorV/Sinopharm was the most common COVID-19 vaccine received (51.2%, *n* = 130), followed by Gam-COVID-Vac/Sputnik-V (22.8%, *n* = 58), and BNT162b2/Pfizer-BioNTech (14.2%, *n* = 36). Interestingly, there was a higher proportion of ChAdOx1-S/nCoV-19/AstraZeneca vaccinated participants with NDPH than non-NDPH (19% vs. 7.9%, *p* = 0.012). Most participants had a complete COVID-19 vaccination scheme (64.6%) and received their last dose between zero and six months before headache onset. Nevertheless, neither vaccination schedule (*p* = 0.377) nor time from the last dose to headache onset (*p* = 0.947) were statistically different between the participants groups (Table 4).

**Table 4:** COVID-19 vaccination status of 421 participants with persistent headache after COVID-19 in Latin America

Vaccination status	All (*n* = 421, 100%)	NDPH (*n* = 106, 25.2%)	Non-NDPH (*n* = 315, 74.8%)	*p* value
Vaccinated against COVID-19 before headache onset, *n* (%)				0.827^a^
No	167 (39.7)	43 (40.6)	124 (39.4)	
Yes	254 (60.3)	63 (59.4)	191 (60.6)	
Type of vaccine, yes (%)				
BBIBP-CorV/Sinopharm	130 (51.2)	28 (44.4)	102 (53.4)	0.217^a^
Gam-COVID-Vac/Sputnik-V	58 (22.8)	9 (14.3)	49 (25.7)	0.062^a^
BNT162b2/Pfizer-BioNTech	36 (14.2)	13 (20.6)	23 (12)	0.09^a^
ChAdOx1-S/nCoV-19/AstraZeneca	27 (10.6)	12 (19)	15 (7.9)	0.012^a^
CoronaVac/Sinovac	16 (6.3)	6 (9.5)	10 (5.2)	0.224^a^
Janssen/Johnson & Johnson	6 (2.4)	3 (4.8)	3 (1.6)	0.164^b^
CIGB-66/Abdala	3 (1.2)	2 (3.2)	1 (0.5)	0.153^b^
mRNA-1273/Moderna	2 (0.8)	0 (0)	2 (1)	1^b^
Ad5-nCoV/CanSino	1 (0.4)	0 (0)	1 (0.5)	1^b^
Vaccination scheme, *n* (%)				0.377^a^
Incomplete (1 dose)	37 (14.6)	9 (14.3)	28 (14.7)	
Complete (2 doses)	164 (64.6)	37 (58.7)	127 (66.5)	
Complete + Booster (≥ 3 doses)	53 (20.8)	17 (27)	36 (18.8)	
Time from the last vaccination dose to headache onset, *n* (%)				0.947^a^
≤ 6 months	213 (83.9)	53 (84.1)	160 (83.8)	
> 6 months	41 (16.1)	10 (15.9)	31 (16.2)	

^a^Pearson’s chi-square test; ^b^Fisher’s exact test

### Factors associated with NDPH

The best valid model (*p* < 0.001, R^2^ Nagelkerke = 0.236, Hosmer–Lemeshow test = 0.711) classified 79.3% (*n* = 334) of participants. Factors associated with higher odds of NDPH were the presence of palpebral edema as a concomitant sign during the acute phase of COVID-19 (OR = 4.945, 95% CI = 1.862–13.13, *p* = 0.001), occipital location (OR = 1.69, 95% CI = 1.016–2.811, *p* = 0.043), and burning character (OR = 2.327, 95% CI = 1.097–4.936, *p* = 0.028). Conversely, onset in the afternoon revealed fewer odds of being associated with NDPH compared to morning onset (OR = 0.246, 95% CI = 0.074–0.821, *p* = 0.023). Finally, attenuating pain showed lower odds of being associated with NDPH (OR = 0.204, 95% CI = 0.078–0.536, *p* = 0.001) (Table 5).

**Table 5:** Factors associated with NDPH in participants with persistent headache after COVID-19 in Latin America

	β	*p* value	OR (95% confidence interval)
Age	0.011	0.307	1.011 (0.99–1.031)
Concomitant symptoms during the acute phase of COVID-19			
Palpebral edema (reference: no)	1.598	0.001	4.945 (1.862–13.13)
Drooping of the upper eyelid and/or pupillary constriction (reference: no)	1.175	0.111	3.238 (0.763–13.735)
Sweating of the face or forehead (reference: no)	0.325	0.255	1.385 (0.79–2.426)
Location			
Occipital (reference: no)	0.525	0.043	1.69 (1.016–2.811)
Character			
Burning (reference: no)	0.845	0.028	2.327 (1.097–4.936)
Onset schedule (reference: during the afternoon)			
During the afternoon	-1.403	0.023	0.246 (0.074–0.821)
During the evening	0.367	0.436	1.443 (0.573–3.631)
At any time of the day	0.101	0.76	1.106 (0.579–2.115)
Attenuating pain (reference: no)	-1.59	0.001	0.204 (0.078–0.536)
Concomitant symptoms of persistent headache after COVID-19			
Paresthesia (reference: no)	0.393	0.158	1.481 (0.859–2.555)
Mental fog (reference: no)	0.46	0.087	1.583 (0.935–2.681)
Nausea (reference: no)	0.501	0.094	1.65 (0.918–2.967)

## Discussion

Among primary headache syndromes, NDPH is one of the most treatment-refractory and difficult-to-diagnose cephalic syndromes. NDPH is a long-term headache with a distinct and clearly-remembered onset and with pain becoming continuous and unremitting within 24 h [16]. Although duration > 3 months is listed among the diagnostic criteria, daily persistence from onset and unremitting headache within 24 h is considered one of the most distinctive features of NDPH. It is primarily bilateral, from moderate to severe intensity [24], and may exhibit features of migraine (unilateral pain, pulsating quality, worsening with physical activity, photophobia, phonophobia, nausea, and vomiting), tension-type headache, or both [7, 14, 25, 26]. The prevalence in the general population is low [27, 28]. However, in patients with chronic daily headache followed at tertiary headache centers, the prevalence ranges from 21 to 28% in pediatric and 2–11% in adult populations [8, 9, 12, 29–31].

Studies of NDPH published before the COVID-19 pandemic reported systemic infections (primarily flu-like syndrome or Epstein-Barr virus infection) as one of the main triggering factors (from 14 to 43% of cases) [25, 26, 32–34]. After the emergence of COVID-19, Trigo López et al. [35] fully characterized 106 patients with acute headache attributed to SARS-CoV-2, of whom 94% met the ICHD-3 criteria for systemic viral infection-related headache. However, these were not classified as NDPH. The first COVID-19-triggered NDPH case was reported by Sampaio Rocha-Filho and Voss in 2020 [12]. Then, more studies of probable NDPH cases in adults with SARS-CoV-2 infection [12, 15] and children and adolescents [18] have followed. The most recent study is a comprehensive multicenter case series from Italy [36].

In this context, we conducted the first study in Latin America exploring the clinical-epidemiological features in a representative cohort of individuals with persistent headache after SARS-CoV-2 infection and the factors associated with NDPH. This study showed that at least a quarter of the participants who had persistent headache after COVID-19 met the criteria for NDPH according to ICHD-3 [16]. Compared to NDPH pre-COVID-19 studies, the mean age and female predominance of participants with NDPH were similar to those reported in India [37], Italy [38], and the USA [24–26, 39]. Conversely, race was determined according to the participant’s self-perception, showing a predominance of mixed and white race, in contrast to other studies showing a predominance of only white race [24–26, 35, 39]. To date, the only large study assessing specifically NDPH and COVID-19 is a case series from Italy [36]. Out of a series of 11 patients with persistent headaches, only eight met the inclusion criteria for NDPH (36.4%) or probable NDPH (36.4%). The remaining three patients with persistent headache were classified as probable migraine (9.1%), and migraine-like headache secondary to COVID-19 (18.2%). All NDPH patients were female, with a median age of 37 years, a median VAS intensity score of 8, and primarily bilateral (90.9%). On follow-up, only three patients (27.3%) remained as NDPH and one (9.1%) as probable NDPH. The remaining patients were reclassified [36]. Among NDPH patients, tension-type-like was the most common pattern while a migraine-like pattern was the most common among probable NDPH patients.

Despite the study’s cross-sectional nature, we identified 106 patients meeting the NDPH/probable NDPH diagnostic criteria. To ensure accuracy in accordance with the NDPH diagnostic criteria, the survey was evaluated by neurologists, infectious disease specialists, and epidemiologists. Participants who did not have a distinct and clearly-remembered onset of headache or did not experience daily headaches were excluded from the study. Additionally, this study did not include individuals with a history of headaches who did not report any change in symptoms after infection with SARS-CoV-2.

While it is known that viral infections may trigger NDPH, the acute infection headache could have been classified as a “headache attributed to infection” (9.2) or even to the subclassification “headache attributed to systemic viral infection” (9.2.2). However, this headache should have significantly improved or resolved in parallel with the improvement in or the resolution of the systemic viral infection (criterion C.3). Also, this headache should not be better accounted for by another ICHD-3 diagnosis (criterion D). In contrast, the type of headache presented by one in four participants in our study is appropriately accounted for by the diagnosis of NDPH (4.10), as they had a persistent headache (criterion A) fulfilling criteria B (distinct and clearly-remembered onset, with pain becoming continuous and unremitting within 24 h) and C (present for > 3 months). Therefore, these diagnoses do not adequately describe participants classified as NDPH. Conversely, the clear line between NDPH, “chronic headache attributed to systemic viral infection”, and long COVID syndrome may be a challenging draw as primary and secondary headache diagnoses may overlap. The ICDH-3 provides guidance on how to choose the most likely diagnosis. Headache is one of the most common neurologic symptoms reported in long COVID [40, 41]. The World Health Organization defines the post-COVID-19 condition or long COVID as new symptoms or the presence of symptoms for over two months, within three months following initial SARS-CoV-2 infection that cannot be explained by any other reason [42]. Long COVID headache and NDPH share a similar clinical picture and their temporal relationship with acute COVID-19, but long COVID headaches may behave as a chronic intermittent headache since they may not be present every day, may respond to analgesics, and may be affected by the severity of the disease [8]. A persistent pattern and sudden onset may more accurately indicate NDPH diagnosis [43]. Fernández-de-Las-Peñas et al. [44] presented a diagnostic model that outlines the distinctive features of NDPH and delayed-onset post-COVID-19 headache in patients with prolonged COVID-19 symptoms. While symptoms such as fatigue, sleep disorders, and anxiety were commonly observed in these patients, the persistent headache clinical characteristics align more closely with those of NDPH.

**Fig. 4:**
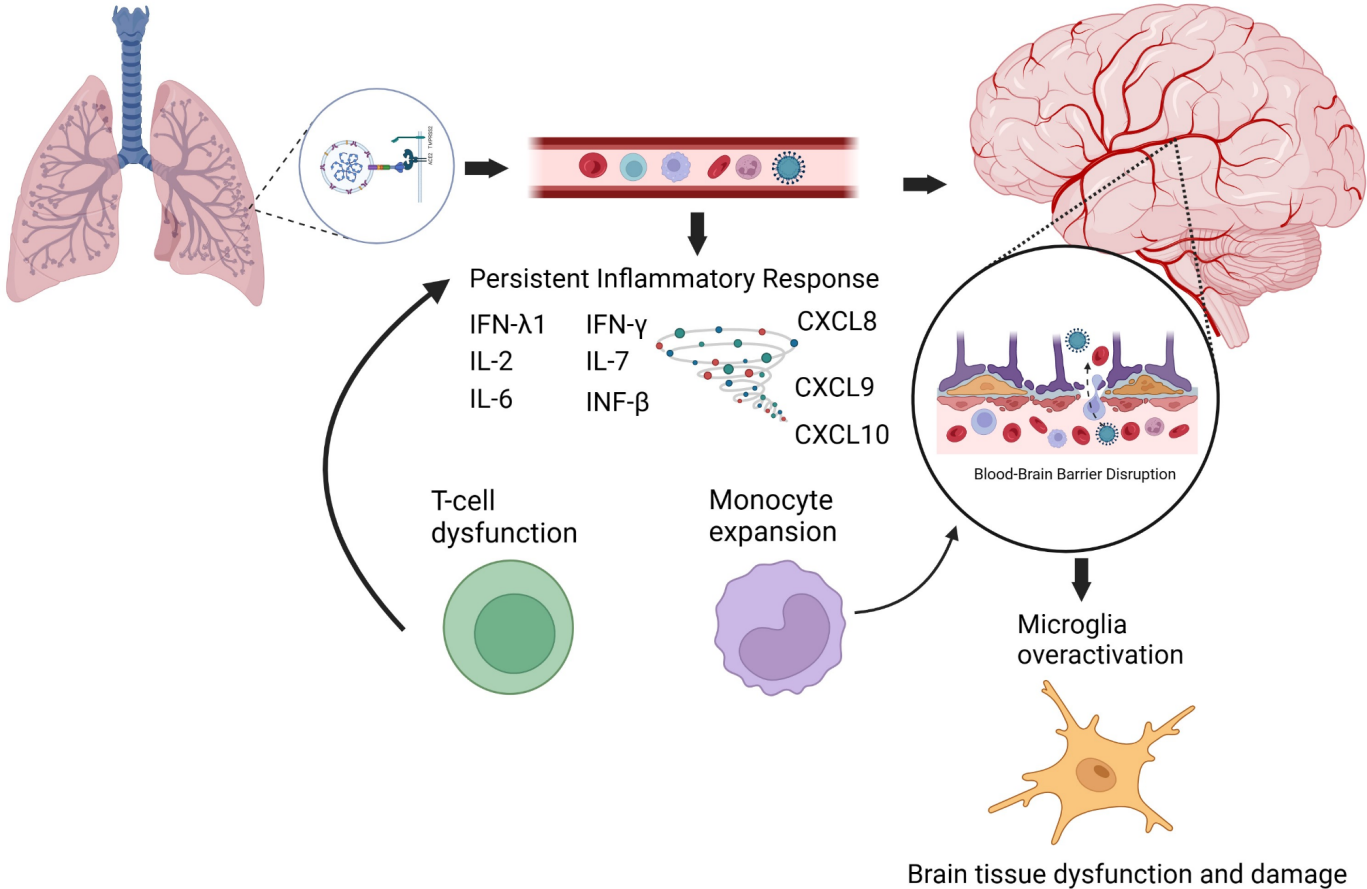
Summary of potential mechanisms involved in the pathogenesis of neurological symptoms in the spectrum of long COVID. Neurological symptoms in long COVID may be attributed to two main mechanisms: viral neuro-invasion and persistent neuroinflammation. The SARS-CoV-2 virus interacts with angiotensin-converting enzyme 2 (ACE2) receptors present not only in the lungs but also in neural cells. One potential route for the virus to enter the central nervous system is through endothelial transcytosis. Notably, the brainstem harbors a significant number of ACE2 receptors, which may account for autonomic dysregulation observed in long COVID patients [46]. Furthermore, patients with neurological symptoms who have recovered from COVID-19 exhibit structural and metabolic brain abnormalities [47–49]. These findings suggest that persistent neuroactivation is a consequence of immunological overactivation triggered by upregulated expression of proinflammatory cytokines such as IFN-β, IFN-λ1, IFN-γ, IL-2, IL-6, IL-17, CXCL8, CXCL9, and CXC10. This immune response activates non-classical and intermediate monocytes, fibroblasts, and myeloid cells while also inducing a dysfunctional T_H_2 cytokine pool that produces CCL11. These pathways collectively result in microglia activation, leading to subcortical white matter demyelination by damaging oligodendrocytes and their precursors, as well as diminished hippocampal neurogenesis. Additionally, T-cell dysfunction and monocyte expansion may contribute to chronic inflammation and disruption of the blood-brain barrier [43]

While mechanisms of NDPH remain largely unknown, several putative mechanisms have been proposed to explain the presence of headache in the spectrum of long COVID. Although clinically different from NDPH, long COVID headache’s potential mechanisms could help guide research into the mechanisms of COVID-19-triggered NDPH (Fig. 4). It is hypothesized that COVID-19 may provoke an exaggerated inflammatory response that overstimulates the trigeminovascular system leading to headaches [10] as occurs in patients with cluster headache, where some assumptions focus on altered neurotransmitter release in this system triggering their attacks [45].

Neuro-invasion has been confirmed in post-mortem brain tissue studies, as evidenced by the presence of ACE2 receptors and type 2 transmembrane serine proteases in neural cells [50, 51]. Most ACE2 receptors have been found in the brainstem, which may explain autonomic dysregulation in long COVID patients [52]. In clinical observational studies, patients with neurological symptoms who recovered from COVID-19 had brain structural and metabolic abnormalities [46, 47, 53]. The mechanism of how SARS-CoV-2 enters the central nervous system remains unclear [40, 48]. However, studies documenting the presence of the virus in neural and capillary endothelial cells suggest endothelial transcytosis as a possible route of access to the central nervous system [49]. Other mechanisms include immunological over-activation leading to persistent neuroinflammation [40, 48]. The ongoing inflammatory response after acute COVID-19 remission keeps the expression of proinflammatory cytokines such as IFN-β, IFN-λ1, IFN-γ, IL-2, IL-6, IL-17, CXCL8, CXCL9, and CXC10 upregulated, the activation of non-classical and intermediate monocytes, fibroblasts, and myeloid cells [40]. Monocyte expansion due to chronic antigen stimulation may lead to blood-brain barrier disruption and neuroinflammation. T-cell dysfunction presents as exhausted CD4^+^ and CD8^+^ and low CD4^+^ central memory, decreasing effector function [40]. Additionally, glial cells become over activated due to high cytokine activity and brain injury. High levels of CCL11 have been found in patients with neurological symptoms of long COVID, inducing microglial migration, leading to hippocampal neurogenesis and ultimately to cognitive dysfunction (mental fog) [40].

Factors predisposing to NDPH remain poorly understood. Consistent with previous studies, COVID-19 clinical severity and the presence of comorbidities were not associated with the development of persistent headache [7, 8]. However, in these studies, persistent headache is not a synonym of NDPH.

While a prior history of migraine has been widely associated with persistent headache after SARS-CoV-2 infection [8, 10], in this study, neither personal history nor family history of migraine were associated with NDPH after COVID-19. Smoking has been identified as a common trigger and intensifier for headaches [54], which is in line with a higher pack-year index found in smokers with NDPH. This makes sense since there is an increased exposure to smoking. Nevertheless, the heterogeneity of NDPH renders comparison between studies challenging and inconclusive when specific subgroups are not studied separately [55].

During the acute phase of COVID-19, patients with persistent headache reported neuropsychological spectrum symptoms more frequently, such as fatigue, sleep problems, anxiety, and mental fog. These symptoms are within the long COVID syndrome, but also are in patients with persistent headache who tend to develop these symptoms more frequently at disease onset [10, 11]. Notably, during the acute phase of COVID-19, a higher proportion of cranial autonomic symptoms were observed in participants with NDPH. These symptoms include sweating of the face or forehead, drooping of the upper eyelid and/or pupillary constriction, and palpebral edema. However, only palpebral edema was identified as a factor associated with higher odds of developing NDPH in the bivariate model. It is also important to clarify that the proportion of cranial autonomic symptoms accompanying NDPH was only evaluated in the univariate analysis. During the development of the bivariate model, these variables were automatically excluded using the Wald method in the backward stepwise variable selection process. Moreover, all cranial autonomic symptoms, with the exception of sweating of the face or forehead, did not exhibit a significantly higher proportion in the NDPH group. The cranial autonomic symptoms described are akin to those seen in trigeminal autonomic cephalalalgias, which include conditions like cluster headache [16] and migraine [56]. Among patients with COVID-19 who were hospitalized due to headache, only 5.8% reported autonomic features [57]. Interestingly, patients with NDPH are generally less likely to have cranial autonomic symptoms associated with their headache [58]. Based on these observations, we propose that cranial autonomic symptoms may not be directly linked to NDPH. Rather, these symptoms might have manifested during the acute phase of COVID-19.

Persistent headache after SARS-CoV-2 infection is a clinically heterogeneous entity. The findings of this study align with previous reports highlighting its oppressive quality and frontal-bilateral topography [11, 59]. Although headache may be located anywhere in the head, in this study, occipital pain was the most common location and was significantly associated with NDPH. Furthermore, the burning character was also associated with NDPH. While the proportion of patients presenting with a burning character was low, and an oppressive-type headache character is more common [6, 18, 19, 26, 60], a burning character may point more specifically to NDPH. Patients who fulfilled the NDPH diagnostic criteria were also more likely to develop headache of severe/unbearable intensity compared to the non-NDPH group; however, disease-associated burden and disability remain high in patients with NDPH [33] and are indistinguishable from those with chronic migraine [61]. In contrast, factors identified that point away from NPDH were afternoon onset periodicity, even though NDPH may present at any time of the day and the presence of factors that attenuate the pain. The latter is evident since NDPH pain is usually refractory to treatment.

Finally, concomitant symptoms in patients with NDPH include sleep disturbances, light-headedness, blurred vision, neck stiffness, concentration problems, sensory disturbances such as numbness or tingling, vertigo, lethargy, and other non-specific syndromes [39]. Mood disorders are considerably more prevalent in patients with NDPH compared to healthy individuals. Moreover, severe anxiety and depressive symptoms were reported in 66% and 40%, respectively, of patients with NDPH [60], findings similar to those of this study, in which a high prevalence of anxious symptoms, sleep problems, and mental fog was observed in patients with NDPH.

This study has several limitations. First, despite receiving responses from 11 Latin American countries, the sample could have been more representative in some countries due to the low number of participants. Furthermore, only Spanish-speaking countries were included, thus excluding Brazil and Haiti, and more than 60% of the responses included were from Venezuelan participants, which may pose a sampling bias. Second, despite the effort to group participants with a diagnosis of NDPH, it was not possible to confirm this diagnosis due to the cross-sectional nature of the study and the absence of neuroimaging to exclude other secondary causes of headache, including meningitis (aseptic or chronic), idiopathic intracranial hypertension, intracranial hypotension, mass lesion, sphenoid sinusitis, hydrocephalus, and cerebral vein thrombosis. However, in most of these clinical instances, imaging studies would also be unrevealing [55]. Furthermore, ICHD-3 diagnoses rely on clinical observations, and probable diagnoses should not be withheld solely due to a lack of ancillary testing. Third, responding to a self-completed online survey carries the risk of participant recall bias, especially if there was a prolonged time between the COVID-19 episode and the survey completion time. Fourth, disseminating the online survey through instant messaging, emails, and social networks could cause selection bias, including only participants with access to smart devices and/or the Internet. Therefore, the findings should be interpreted with caution. Finally, since this is a cross-sectional study, follow-up data is unavailable. As a result, some participants’ clinical conditions may have changed over time, requiring their diagnosis to be reclassified in the future. Nevertheless, the study’s strengths are the relatively diverse study population included, a wide variety of vaccine types reported by the participants, and a systematic way of data collection that prevented missing data or participants not meeting the inclusion criteria from being included. We believe the findings may provide valuable information that could serve as the background for future research to aid the diagnosis of this complex cephalic syndrome.

## Conclusion

The prevalence of post-COVID-19 headache is a growing concern, as it may persist for several months and be unresponsive to treatment. Therefore, healthcare workers must consider NDPH as a possible diagnosis in COVID-19 patients with persistent headaches. It is crucial to optimize patient evaluation and follow-up through post-COVID-19 consultations, especially in Latin American countries. Conducting multicenter and longitudinal studies on NDPH after COVID-19 may provide a better understanding of its clinical evolution. In addition, raising patient awareness of NDPH and training physicians on the clinical, diagnostic, and treatment of this pathology may improve the quality of life of those who suffer from it.

### Electronic supplementary material

Below is the link to the electronic supplementary material.

Supplementary Material 1

## Data Availability

All data and materials in this article are included in the manuscript.

## Abbreviations

COVID-19: Coronavirus disease 2019
SARS-CoV-2: Severe acute respiratory syndrome coronavirus 2
NDPH: New daily persistent headache
RT-PCR: Reverse transcriptase polymerase chain reaction
VAS: visual analogue scale
ICHD-3: Third edition of the International Classification of Headache Disorders
SD: Standard deviation
IQR: Interquartile range
OR: Odds ratio
CI: Confidence interval
ACE2: Angiotensin converting enzyme 2
