# Supplementary material for: New daily persistent headache after SARS-CoV-2 infection in Latin America: a cross-sectional study
Source: BMC Infect Dis. 2023 Dec 14;23:877. doi: 10.1186/s12879-023-08898-2 (PMC10722794; doi:10.1186/s12879-023-08898-2)
Supplement: Supplementary file 1 — Supplementary Material 1 [file 12879_2023_8898_MOESM1_ESM.docx]

Post-COVID-19 headache survey in Latin America

Dear participant,

Venezuelan researchers from the Institute for Biomedical Research and Therapeutic Vaccines (VACTER) and the Venezuelan Science Incubator (IVC) are conducting a study on the characteristics of COVID-19 related headaches in Latin America. Your participation is completely voluntary, anonymous, and confidential, and will help us to better understand this important pathology. At the end of the survey, you will be able to download a free document with recommendations from Neurology and Infectious Diseases physicians and neuroCOVID experts that can help you alleviate your headaches.

We thank you very much for your contribution to science by answering a total of 37 questions that we prepared for this purpose and agreeing to participate in this study. Answering or not answering the questions will be of no consequence to you. If you understand the purpose of this study, agree to participate, and are over 18 years of age, please start the survey.

* Indicates that the question is mandatory

# 1. ¿Have you had COVID-19? *

## Mark only one oval.

Yes *Skip to question 2*

No *Skip to section 7 (Thank you very much for your participation!)*

# 2. Have you had a headache during and/or after COVID-19? *

## Mark only one oval.

Yes *Skip to question 6*

No *Skip to section 7 (Thank you very much for your participation!)*

# If the answer above was "Yes", please state how long the headache lasted or has lasted.

## Mark only one oval.

Less than 4 weeks *Skip to section 7 (Thank you very much for your participation!)*

4 weeks or more *Skip to question 6*

# If the answer above was "Yes", please state how you were diagnosed with COVID-19 on that occasion.

## Mark only one oval.

I had a nasopharyngeal swab sample taken and it was positive (PCR or rapid antigen test).

I had a blood sample taken and it came back positive (rapid antibody test).

I was diagnosed by a physician because of my symptoms, abnormal chest images, and/or low oxygen saturation (clinical)

I self-diagnosed because someone in my family was diagnosed with COVID-19 and we had similar symptoms

# If the previous answer was "Yes", tell us what was your clinical evolution on that occasion when you presented COVID-19.

## Mark only one oval.

I had no symptoms (asymptomatic)

I had symptoms but did not have shortness of breath or abnormal chest images (mild)

I had shortness of breath or abnormal chest images and an oxygen saturation ≥94% (moderate)

I was hospitalized or treated at home for an oxygen saturation <94% and an oxygen requirement (severe)

I was hospitalized in intensive care or treated at home with mechanical ventilation (critical)

# 3. Which of the following symptoms did you have BEFORE the COVID-19 pandemic? *

You may check more than one option

*Select all that apply.*

Deep sadness, depression, and loss of interest in things (depressive symptoms)

Nervousness, restlessness, anxiousness, tension, and/or palpitations (anxious symptoms)

Sleep problems

None of the symptoms previously mentioned

# 4. Did you suffer from any type of headache BEFORE the COVID-19 pandemic? *

You may check more than one option

*Select all that apply.*

Yes, migraines

Yes, headache due to muscle contraction (tensional)
I did not suffer from any type of headaches previously

Other:

# If the above answer was "Yes", who diagnosed or told you that type of headache you had?

## Mark only one oval.

A specialist in Internal Medicine or Neurology

A general physician

Family/Friends/Neighbor Self-diagnosed

Other:

# If the answer above was "Yes", tell us how that type of headache evolved compared to the one that lasted or has lasted 4 weeks or more

## Mark only one oval.

It was or is the same headache but it got better or has gotten better

It was or is the same headache and it stayed or has stayed the same

It was or is the same headache but it got worse or has gotten worse

It was or is different headaches

# 5. Which of the following symptoms did you have DURING the first 4 weeks when you presented with COVID-19? *

You may check more than one option

*Select all that apply.*

Feeling that the whole body or some part of the body is bigger or smaller than it really is (total or partial body macrosomatognosia or microsomatognosia)

Perception that things are larger or smaller than they really are (macropsia or micropsia)

Perception that things are farther away or closer than they really are (teleopsia or pelopsia)

Feeling that the world or oneself is unreal (derealization or depersonalization)

Profound sadness, depression, and loss of interest in things (depressive symptoms)

Total or partial inability to perceive colors (achromatopsia or hypochromatopsia)
Ear discomfort from loud sounds (noise sensitivity or phonophobia)
 Smell discomfort from strong odors (odor sensitivity or osmophobia)
Tingling, numbness, or prickling in any part of the body (paresthesia)
Nervousness, restlessness, anxiousness, tension, and/or palpitations (anxious symptoms)
Sensation of time speeding up or slowing down (time distortion)
Perception that lines and contours are wavy (dysmorphopsia)

Increased volume of the upper and/or lower eyelid (eyelid edema)
Reduced attention, concentration, and memory (confusion or mental fog)
 Eye discomfort from bright light (light sensitivity or photophobia)
Perception that colors are very bright (hyperchromatopsia)
Loss of consciousness or awareness (fainting)

Drooping of the upper eyelid and/or constriction of the pupil
Total or partial loss of taste (ageusia or hypogeusia)
Total or partial loss of smell (anosmia or hyposmia)
 Whistling or ringing in one or both ears (tinnitus)
Sensation of floating in the air (illusory levitation)

Persistent bad taste in the mouth (dysgeusia)
 Color confusion (dyschromatopsia)
Tiredness or exhaustion (fatigue)
Sweating of face or forehead

Muscle aches (myalgia)
Red vision (erythropsia)
Sleep problems
Nasal congestion

Tearing
 Nausea
Vomiting
Dizziness
Fever

I did not have any of these symptoms

# 6. Do you currently have any of the following diseases? *

You may check more than one option

*Select all that apply.*

Hypertension

Chronic obstructive pulmonary disease (COPD) Diabetes

Human Immunodeficiency Virus (HIV) Cancer

Chronic kidney disease (CKD) Asthma

I do not have any disease

Other:

# 7. Do you currently smoke? *

## Mark only one oval.

Yes
No

# If the answer above was "Yes", how many cigarettes do you smoke per day?

1. If the answer above was "Yes", how many years have you been smoking?

# 8. Do you have a family member (parent or sibling) diagnosed with any type of headache BEFORE the COVID-19 pandemic? *

You may check more than one option

*Select all that apply.*

Yes, migraines

Yes, headache due to muscle contraction (tensional)
I do not have any family members suffering from any type of headaches

Other:

Next, we will ask you some questions related to the headache that lasted or has lasted 4 weeks or more during and/or after COVID-19.

1. 9. When did the headache start? *

## Mark only one oval.

Within the first 2 weeks of the start of COVID-19

Between the 2nd and 4th week after the start of COVID-19 After the 4th week after the start of COVID-19

# 10. Do you clearly remember the day when the headache started? *

## Mark only one oval.

Yes

No

# If the above answer was "Yes", state the exact date when the headache started.

*Example: January 7, 2019*

# If you do not remember the exact date, at least state the month and year when the headache began.

For example: November 2021

# 11. How did the headache start? *

## Mark only one oval.

Abruptly/rapidly (sudden)

Progressive/slow form (insidious)

# 12. Where was or is the headache located? *

## Mark only one oval.

Right half or left half of the head (unilateral)
 Whole head (bilateral)

# 13. Where was or is the headache located? *

You may check more than one option

*Select all that apply.*

On the top right and/or top left side of the head (parietal)

At the front of the head (frontal)

At the back of the head (occipital)

At the top of the head (vertex)

At the side of the head (temporal)

At the eyes (periocular)

Visual Analog Scale (VAS) for Pain


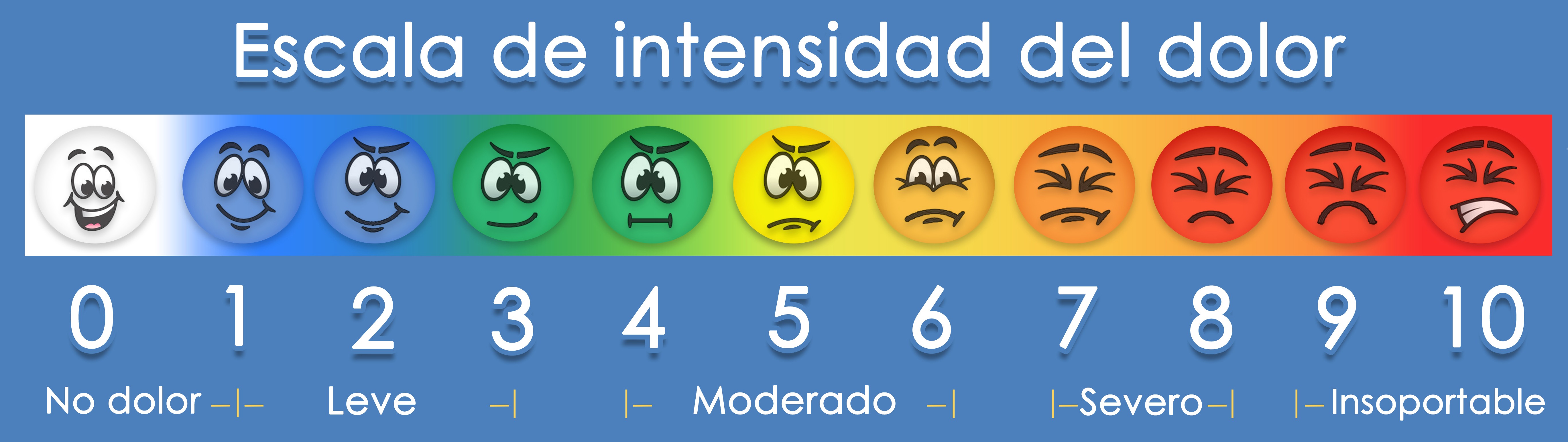


1. 14. According to the VAS for pain, how severe was or is the headache? *

## "No pain" (0 points) means the headache does not affect your daily activities; "Mild" (1, 2 and 3 points) means it affects less than half of them; "Moderate" (4, 5 and 6 points) means it affects half of them; "Severe" (7 and 8 points) means it affects more than half of them; and "Unbearable" (9 and 10 points) means the headache makes you bedridden.

## Mark only one oval.

0

1

2

3

4

5

6

7

8

9

10

# 15. How was or is the type of headache? *

You may check more than one option

*Select all that apply.*

Throbbing sensation (pulsating) Compression sensation (oppressive)

Burning sensation (burning or stinging)

Mild but continuous pain that sometimes increases (dull)
Sudden prickling sensation (lancinating or stabbing)

# 16. To what part did or does the headache extend? *

You may check more than one option

*Select all that apply.*

Face
Neck
Shoulders

Headache does or did not spread

Other:

# 17. How long did your headache last or has it lasted? *

## Mark only one oval.

Between 4 and 5 weeks

Between 5 and 6 weeks

Between 6 and 7 weeks

Between 7 and 8 weeks

Between 8 weeks and 3 months

Between 3 and 6 months

Between 6 and 9 months

Between 9 months and 1 year
 More than 1 year

# 18. How often did or do you get headaches? *

## Mark only one oval.

Daily

Interdaily
Twice a week
Once a week

Once every two weeks Once a month

# 19. Specify how many times a month you had or have a headache *

1. 20. At what time of the day did or does your headache start? *

## Mark only one oval.

In the morning
In the afternoon
In the night
At any time

# 21. How were or are you relieved of your headache? *

You may check more than one option

*Select all that apply.*

Sleeping
Resting

Taking painkillers such as acetaminophen, ibuprofen, diclofenac, ketorolac, etc. Nothing relieves or relieved my headache

Other:

# If something relieves or relieved your headache, please tell us in what percent

## Mark only one oval.

0%

10%

20%

30%

40%

50%

60%

70%

80%

90%

100%

# 22. What aggravated or aggravates your headache? *

You may check more than one option

*Select all that apply.*

Coughing, sneezing, bending over and/or tilting your head
Moving your head

Walking

The headache does or did not get worse

Other:

# 23. Which of the following symptoms accompanied or accompany the headache? *

You may check more than one option

*Select all that apply.*

Feeling that the whole body or some part of the body is bigger or smaller than it really is (total or partial body macrosomatognosia or microsomatognosia)

Perception that things are larger or smaller than they really are (macropsia or micropsia)

Perception that things are farther away or closer than they really are (teleopsia or pelopsia)

Feeling that the world or oneself is unreal (derealization or depersonalization)

Profound sadness, depression, and loss of interest in things (depressive symptoms)

Total or partial inability to perceive colors (achromatopsia or hypochromatopsia)
Ear discomfort from loud sounds (noise sensitivity or phonophobia)
 Smell discomfort from strong odors (odor sensitivity or osmophobia)
Tingling, numbness, or prickling in any part of the body (paresthesia)
Nervousness, restlessness, anxiousness, tension, and/or palpitations (anxious symptoms)
Sensation of time speeding up or slowing down (time distortion)
Perception that lines and contours are wavy (dysmorphopsia)

Increased volume of the upper and/or lower eyelid (eyelid edema)
Reduced attention, concentration, and memory (confusion or mental fog)
 Eye discomfort from bright light (light sensitivity or photophobia)
Perception that colors are very bright (hyperchromatopsia)
Loss of consciousness or awareness (fainting)

Drooping of the upper eyelid and/or constriction of the pupil
Total or partial loss of taste (ageusia or hypogeusia)
Total or partial loss of smell (anosmia or hyposmia)
 Whistling or ringing in one or both ears (tinnitus)
Sensation of floating in the air (illusory levitation)

Persistent bad taste in the mouth (dysgeusia)
 Color confusion (dyschromatopsia)
Tiredness or exhaustion (fatigue)
Sweating of face or forehead

Muscle aches (myalgia)
Red vision (erythropsia)
Sleep problems
Nasal congestion

Tearing
 Nausea
Vomiting
Dizziness
Fever

I did not have any of these symptoms

# 24. Who have you seen for evaluation and/or treatment for your headaches? *

*Select all that apply.*

A specialist in Internal Medicine or Neurology
A general physician

A family member/friend/neighbor

I have not been to anyone for evaluation and/or treatment of my headache.

Other:

# 25. Do you STILL have a headache? *

## Mark only one oval.

Yes

No

# 26. What was or has been the evolution of your headache? *

## Mark only one oval.

It has improved partially or completely

It went on or stayed the same
It got or has been gotten worse

# 27. BEFORE the onset of the headache that lasted or has lasted 4 weeks or more, were you vaccinated against COVID-19? *

## Mark only one oval.

Yes
No

# If the answer above was "Yes", what type of vaccines did you have?

You may check more than one option

*Select all that apply.*

Sputnik-V Sinopharm AstraZeneca Sinovac/CoronaVac Johnson & Johnson Moderna

Pfizer

Other:

# If the answer above was "Yes", how many doses of the vaccines did you have?

## Mark only one oval.

1

2

3

4

Other:

1. If the answer above was "Yes", how many months BEFORE you had your last dose?

## Mark only one oval.

Less than 3 months
Between 3 and 6 months

Between 6 and 9 months

Between 9 months and 1 year
 More than 1 year

# 28. How many times have you had COVID-19? *

## Mark only one oval.

- 1. time
  2. times
  3. times
  4. times

Oher:

# 29. Indicate the month and year of each time you have gotten COVID-19 *

For example: 1st time in June 2020, 2nd time in January 2021, 3rd time in November 2021

1. 30. In which episode of COVID-19 did the headache that lasted or has lasted 4 weeks or more begin? *

## Mark only one oval.

On the 1st time

On the 2nd time

On the 3rd time

On the 4th time

# 31. How old are you? *

1. 32. What is your gender? *

## Mark only one oval.

Female Male

# 33. What is your highest level of education? *

## Mark only one oval.

None

Elementary School
High School

Technician

University

1. 34. What is your marital status? *

## Mark only one oval.

Single

Cohabitant
 Married

Divorced Widowed

# 35. What is your race? *

## Mark only one oval.

Mestizo (mulatto/brown) White (Caucasian)
Black (African-American) Indigenous (native)

# 36. What is your occupation? *

## Mark only one oval.

I work full-time or part-time (employed)
I work in health care (health care personnel)
I am self-employed (independent)

I am unemployed/retired
I am a student

# If you work or study, please specify the field

For example: Secretary, Medical Internist, Merchant, Engineering Student.

# If you work or study, state the number of hours per day.

## Mark only one oval.

Less than 4 hours
 Between 4 and 8 hours

Between 8 and 12 hours

More than 12 hours

# If you work or study, please state your type of workday

## Mark only one oval.

Daytime Nighttime Mixed

# 37. In which Latin American country do you live? *

## Mark only one oval.

Argentina Belize Bolivia Chile Colombia Costa Rica Cuba Ecuador

El Salvador Guatemala Honduras México Nicaragua Panamá Paraguay Perú Puerto Rico

Dominican Republic Uruguay

Venezuela

Other:

# If you wish, you can leave us your cell phone number in case we find a speciﬁc diagnosis of headache and we can provide you with support

For example: +58 (414) 9347456

Thank you very much for your participation!

Click "Submit" to access the link with recommendations from Neurology and Infectious Diseases physicians and neuroCOVID experts.

This content has not been created or approved by Google.

[Forms](https://www.google.com/forms/about/?utm_source=product&utm_medium=forms_logo&utm_campaign=forms)
